# Pulmonary tuberculosis in Romania at the dawn of the  millennium–a major public health issue

**Published:** 2009-04-25

**Authors:** C Marica

**Affiliations:** ‘Marius Nasta’ Institute of Pneumology, BucharestRomania

## Abstract

TB incidence in our country is still quite high compared to the average of the European Union countries (1^st^ place among EU countries and 3^rd^ place among WHO European Region countries), which means that a national coordinated response against this disease needs to become the priority of the current health care policy.

The multi–factorial conditioning, which includes the social and economic dimensions of TB spreading, requires a multi–disciplinary and inter–sectorial approach to this pathology, going beyond healthcare services.

The National Tuberculosis Control Strategy is a part of Romania's Country Strategy based on the guidelines set out in WHO's 2006–2015 Global Plan to Stop Tuberculosis (MDGs 2015) and it provides the necessary framework for refining and harmonizing the national legislation and regulations with the European laws after Romania's integration in the EU.

In 1993, the World Health Organization declared tuberculosis ‘to be a global health emergency’. The annual reports of the World Health Organization (WHO) indicate that there are approximately 9 million new TB cases and 2 million TB–related deaths in the world, every year. Over 80% of the patients come from Asian and Sub–Saharan African countries [1, 2] Actually, compared to previous years, the number of TB cases is the same or has dropped in 5 of the 6 WHO regions, but it has increased in Africa, mainly due to the constant spreading of HIV infection [6,7,8,9]

Taking into account the European context, even after Romania has integrated in the EU, the TB incidence in our country is still quite high compared to the average of the European Union countries (1^st^ place among EU countries and 3^rd^ place among WHO European Region countries). This means that a national coordinated response against this disease needs to become the priority of the current health care policy.

The multi–factorial conditioning, which includes the social and economic dimensions of TB spreading, requires a multi–disciplinary and inter-sectorial approach to this pathology, going beyond healthcare services.

After adopting the International Direct Observation Treatment Strategy (DOTS) in 1998 and implementing the 1997–2000 and 2001–2005 National Tuberculosis Control Programs (NTP), in 2003 Romania recorded, for the first time in the last 20 years, a drop in the new TB cases. These promising results are due to more intense NTP activities and to the implementation of projects financed by the World Health Organization and the Global Fund to Fight HIV/AIDS, Tuberculosis, and Malaria.

Thus, with an average therapeutic success rate for the new 82% bacteriological confirmed cases, Romania is getting closer to the global target of 85% [3,10].

Nevertheless, in 2006, a number of 25,422 new and relapsed tuberculosis cases (117.8 cases per 100,000 inhabitants) were recorded in Romania, which unfortunately placed our country among the first in Europe.

The National Tuberculosis Control Strategy is part of Romania's Country Strategy based on the guidelines set out in WHO's 2006– 2015 Global Plan to Stop Tuberculosis (MDGs 2015) and it provides the necessary framework for refining and harmonizing the national legislation and regulations with the European laws after Romania's integration in the EU.

The incidence of this disease continuously increased in Romania, from 55.8%ooo in 1985 to 142.2%ooo in 2002. The deteriorated social and economic background and the dysfunctions in the operation of the healthcare system caused this problem. [3,4]

In 2003, for the first time in the last 20 years, a slight decline of the annual TB incidence rate was recorded in Romania, namely 135.7%ooo as compared to 142%ooo in 2002. This decline is the first sign showing that all the last years' efforts to control tuberculosis in Romania are starting to get good results. Still, because this is a biological phenomenon, more time is needed and sustained interventions and activities must be carried out before we can say, without any doubt that the incidence rate has dropped. Actually, the rise of the global tuberculosis incidence has the tendency ‘to stop’; this is confirmed by the values recorded in 2005 and 2006, when the global tuberculosis incidence rate continued to drop and reached 123.7%ooo and 116.8%ooo, respectively, and a further more 109,8%ooo in 2007 [5,6,7,8].

**Fig 1:**
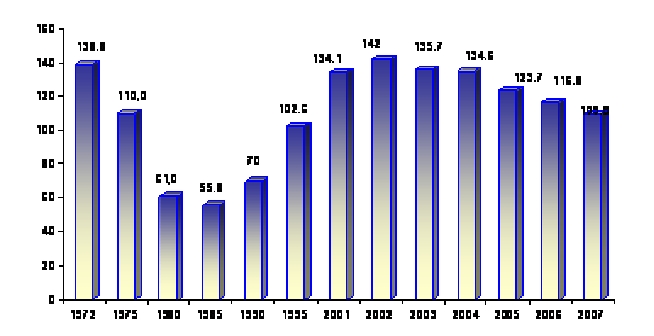
Evolution of tuberculosis' global incidence in Romania between 1972 and 2007

The highest incidence rate is recorded in the cases of 22 to 55 year–old people.

The TB incidence in children has continuously increased, from 7.9%ooo in 1985 to 48.2%ooo in 2002. The TB incidence in children has adopted the same decreasing trend starting with 2003 (44.3%ooo), then in 2004 (41.2%ooo), 2005 (33.4%ooo), 2006 (31.8%ooo) and 2007 (28.7%ooo)(Fig 2).

**Fig 2:**
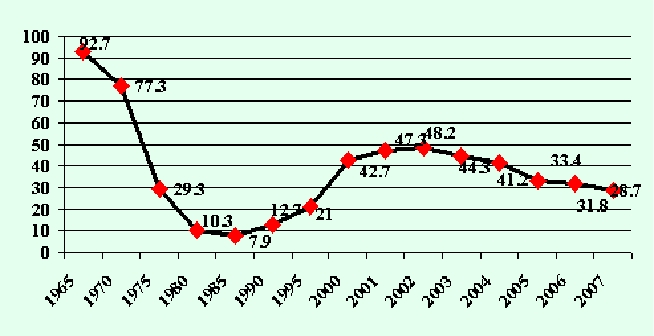
Evolution of TB incidence in children between 1972 and 2007

The Program became a success thanks to a functional well–defined management structure. The Central Management Unit (CMU) at ‘Marius Nasta’ Institute of Pneumology managed it.

The line management of the program was the responsibility of the Public Health Department and 50 managers (or county coordinators–41 county managers, 6 managers for each of the Bucharest districts and 3 managers representing the Ministry of Justice, the Ministry of Defense, and the Ministry of Interior). There are 3 to 6 TB dispensaries in each county – 189 in total and the specialized medical network is made up of approximately 900 pneumologists.

General practitioners (GP) were also involved in NTP, especially in rural areas, as they were in charge of screening tuberculosis suspects and administering direct observation treatment (DOT).

There is at least one hospital addressing TB patients (76 TB and lung diseases hospitals with approximately 7,000 beds for TB) in each county.

In Romania, a quite original structure was set up–The National Commission for Program Supervision–made up of 30 pneumology specialists trained for this work, whose role is to watch if the Program provisions are where they are supposed to be at national level.[10]

The diagnosis and treatment is free for all TB patients. All costs are covered by the health insurance social funds and the state budget for preventive medicine and public health. A number of 190 hospital pharmacies provide access to medication.

Currently, a great importance is given worldwide to multidrug–resistant tuberculosis (MDR–resistant to at least isoniazid and rifampicin). Moreover, a lot of attention is also paid to the need of running an overall assessment of the phenomenon, insisting on XDR tuberculosis (resistant to the two major drugs (H and R) plus any fluoroquinolone and to at least one of the three second–line injection drugs (Capreomicyn, Kanamycin and Amikacin) [2,10].

In 2005, a report on Surveillance of Drug Resistance in Tuberculosis indicated a 2.8 % MDR–TB rate of new cases and a 11.6 % rate of relapsed cases. These data place our country below the other Eastern European and former Soviet Union countries (new cases: in The Russian Federation–15.5%; Kazakhstan–28%; The Republic of Moldova–18.8%; Lithuania–20%; Bulgaria–5.7%) [9].

The latest drug–resistance survey carried out in our country took place in 2003 and 2004, with the technical support of WHO. Compared to the global situation, the MDR TB is not an extremely serious problem for Romania as its incidence rate is very close to the global average. Nevertheless, with approximately 22,000 new TB cases each year, we estimate that the number of new MDR TB patients will increase to over 600 yearly [8].

The recent increase of drug–resistant BK strains incidence and mainly of MDR TB requires a closer national and international monitoring of this phenomenon. At a national level, we already have two MDR TB treatment and monitoring centers of excellence (in Bucharest and Bisericani), whose evaluation (run approximately three years after their opening) is quite promising.

As far as the territorial application of the NTP provisions is concerned, undoubtedly there are still some dysfunctions related to the obsolescence of the radiology equipment, to inappropriate locations and insufficient medical staff in TB dispensaries. We hope these problems will soon be tackled, before they set a negative impact on the TB prevention, screening, and treatment work.

After the Romanian Government and the Global Fund to Fight HIV/AIDS, Tuberculosis and Malaria a grant agreement was signed in 2004. The work of NTP has also been supported by TB projects carried out under this agreement.

In conclusion, the existence of an efficient TB network at a national level of a well–implemented National Program, the involvement of pneumologists, epidemiologists and general practitioners in this matter, and mainly the financial and moral support of the Romanian Government and that of the Ministry of Public Health are necessary starting points in order to meet the criteria of WHO's current STOP TB Plan.
